# In Case of War

**Published:** 1898-04

**Authors:** 


A Monthly Review of Medicine and Surgery.

*EDITORS:*

THOMAS LOTHROP, M. D. - - WM. WARREN POTTER, M. D.

All communications, whether of a literary or business nature, should be addressed to the managing editor: 284 FRANKLIN STREET, BUFFALO, N. Y.

VOL. XXXVII. APRIL, 1898. No. 9.

### IN CASE OF WAR.

WHILE it is to be hoped that the probabilities of war with Spain are yet remote, it is still fitting that the government should be prepared for every emergency that can possibly arise. In the present strained conditions between the two governments it is possible that a new turn to affairs may be presented at any moment. The activity in naval preparations on every hand give token that the administration at Washington is fully alive to the situation. The army, too, is displaying commendable alertness with a view to the adequate defense of our extensive and greatly exposed seacoast.

In the midst of all this energetic preparation it becomes the medical department of both arms of the service to be well organised for active duty on sea and land. Fortunately, there are men of large experience presiding over the medical departments of the army and navy who have seen active service in our late civil war. They have kept pace with all modern improvements relating to the care of sick and wounded and are, therefore, especially fitted to cope with any emergency that can possibly present itself.

It is highly probable in case of war with Spain that the principal fighting will take place upon the sea. Nevertheless, there is a large opportunity for active service on land in the way of erecting and manning defenses all along the coast from Maine to Texas in the East and South, not to speak of California, Oregon and Washington in the West. A large number of medical officers will be needed to care for these troops, and it becomes every medical man fitted for such service to keep himself well prepared for any call that may be made upon him.

There are still living many competent surgeons who served in the civil war who possess excellent organising qualifications, and whose services could be utilised to advantage by the government in the circumstances under contemplation. The experience of such men would be valuable in many ways in putting a new force into the field, details of which we need not now take time nor space to mention, our object simply being to call attention to the general fact.

It is quite true that many of the conditions governing field hospital organisation have materially changed since 1865, yet there are great underlying principles which still hold good. A well-trained, educated civil-war surgeon who has kept pace with events could not fail to be useful at the present time, and we have no doubt there are many such who are yet stimulated with a high spirit of patriotism, and who would gladly enter the service again should necessity arise.

Surgeon-general Sternberg is a man most admirably equipped to direct the medical affairs of the army and it is most fortunate that circumstances have placed him in his present position at this time. He well knows war and war's needs and his eminent scientific attainments command the respect and confidence, not only of the government, but of the medical profession of the civilised world. As president of the American Medical Association this year he will be brought in touch with American physicians of prominence from every quarter of the Union—a circumstance of no mean import at this juncture.

The army, too, is to be congratulated in having at its head major-general Nelson A. Miles, a man who has served in every capacity in which musket and saber are used and who has participated in more active campaigns than any other living soldier of eminence. He is in the very prime of a magnificent manhood and whether as strategist or tactician he has no superior in any country as a superbly-equipped army commander.

Finally, therefore, taken all in all we are in splendid condition for offensive or defensive warfare, and a patriotic people full of energy, inventive genius, financial strength, headed by a well-balanced, capable, statesmanlike executive in the person of President McKinley, may confidently expect a brilliant and successful issue in any undertakings that may become necessary to clear the atmosphere of the present shadow which overhangs our relations with Spain.

